# MiR-199a-5p and let-7c cooperatively inhibit migration and invasion by targeting MAP4K3 in hepatocellular carcinoma

**DOI:** 10.18632/oncotarget.14623

**Published:** 2017-01-13

**Authors:** Lili Liu, Liqin Lu, Aihong Zheng, Jiansheng Xie, Qian Xue, Fuwei Wang, Xiao Wang, Hongying Zhou, Xiangmin Tong, Yaqing Li, Xiuming Zhu, Guoqing Wu

**Affiliations:** ^1^ Department of Oncology, Sir Run Run Shaw Hospital, College of Medicine, Zhejiang University, Hangzhou, China; ^2^ Department of Oncology, Zhejiang Provincial People's Hospital, Hangzhou, Zhejiang, China; ^3^ Department of Hematology, Zhejiang Provincial People's Hospital, Hangzhou, Zhejiang, China; ^4^ Department of Respiratory, Zhejiang Provincial People's Hospital, Hangzhou, Zhejiang, China

**Keywords:** hepatocellular carcinoma, miR-199a-5p, let-7c, MAP4K3, metastasis

## Abstract

Hepatocellular carcinoma (HCC) has a high recurrence rate, and patients exhibit poor survival mainly because intrahepatic metastasis is common. We previously reported that let-7c down-regulation is significantly associated with poor differentiation level in HCC. In the present study, we demonstrate that miR-199a-5p and let-7c are frequently down-regulated in HCC cells and tissues, and low expression of miR-199a-5p is correlated with tumor size, liver envelope invasion. Furthermore, miR-199a-5p and let-7c cooperatively inhibit HCC cell migration and invasion *in vitro*. MAP4K3 is identified as the direct target of miR-199a-5p and let-7c and this regulation is further confirmed by luciferase reporter assays and Western blotting. In addition, MAP4K3 functions as a metastasis promoter since the results demonstrate that MAP4K3 could promote HCC cell migration and invasion. We also find that miR-199a-5p and let-7c increase the sensitivity of HCC cells to sorafenib.

**Conclusions:**

We report that miR-199a-5p and let-7c cooperatively and efficiently inhibit HCC cell migration and invasion by targeting the metastasis promoter MAP4K3 and MAP4K3-mediated drug sensitization, suggesting that the use of miRNAs and sorafenib in combination therapy may be a powerful approach to the treatment of HCC.

## INTRODUCTION

Hepatocellular carcinoma (HCC) is a leading cause of cancer death in many Asian and African countries. HCC causes approximately 662,000 deaths each year worldwide, and approximately 55% of cases occur in China [1–3]. The pathogenesis of HCC is a multistage process that is typically associated with chronic liver inflammation, liver cirrhosis or alcohol abuse [4, 5]. Despite great advances in screening for the disease, HCC is often diagnosed at an advanced stage, at which it does not respond to radical treatment. Owing to the high rate of recurrence and metastasis, advanced HCC has a poor prognosis and a high fatality rate. Therefore, the identification of new molecules involved in tumor metastasis is a key step toward the development of novel therapeutics.

MicroRNAs (miRNAs) are a class of highly conserved non-protein-encoding short RNA molecules that regulate genes at the post-transcriptional level [6]. Emerging evidence has indicated that many miRNAs are dysregulated in HCC and that some specific miRNAs are associated with metastasis, recurrence, and prognosis [7–9]. Moreover, some miRNAs, such as miR-122, miR-21, miR-192, play important roles in regulating HCC growth, apoptosis, migration and invasion [10–12]. The microRNA let-7 acts as a tumor suppressor by inhibiting Ras oncogenes [13]. Takamizawa et al. reported that reduced expression of let-7 in human lung cancer was associated with a shorter postoperative survival [14]. Han and colleagues demonstrated that let-7c functions as a metastasis suppressor in colorectal cancer through its targeting of MMP11 and PBX3 [15]. Most of important, there was a report showed that let-7c induced apoptosis in HCC in cooperation with an anti-cancer drug sorafenib [16]. Shen et al. demonstrated that miR-199a-5p was consistently decreased in HCC tissues and cell lines, and low miR-199a-5p expression contributes to increased cell invasion by deregulation of DDR1 activity in HCC [17]. These studies suggest that let-7c and miR-199a-5p play a crucial role in the growth and development of liver cancer. However, the impact of let-7 and miR-199a on HCC metastasis has received minimal attention.

In our previous study, we found that let-7c expression was significantly decreased in HCC tissues, and a correlation was noted between the decrease in let-7c expression and poor differentiation level in HCC [18]. In this study, we comprehensively investigated the biological functions and underlying molecular mechanism of let-7c and miR-199a-5p in HCC metastasis. The enhanced expression of miR-199a-5p and let-7c significantly inhibited HCC cell migration and invasion. Interestingly, miR-199a-5p and let-7c exhibited combinatorial effects on the decrease in HCC cell migration and invasion. Furthermore, MAP4K3, a putative metastasis promoter in HCC, was characterized as a direct and functional target of let-7c and miR-199a-5p. miR-199a-5p and let-7c increased the sensitivity of HCC cells to sorafenib. These results indicate a possible regulatory pathway involving mitogen-activated protein kinases (MAPKs) and a candidate target for HCC treatment.

## RESULTS

### Expression of miR-199a-5p and let-7c is decreased in HCC cell lines and tissues and is associated with HCC metastasis

**Figure 1:**
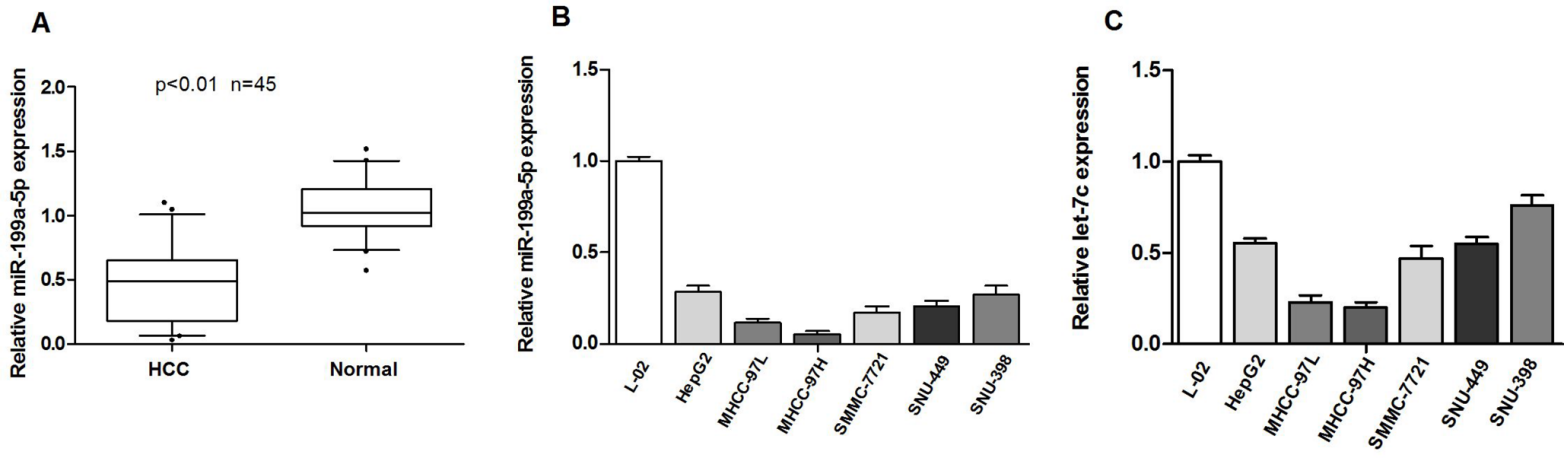
Expression of miR-199a-5p and let-7c is decreased in HCC cell lines and tissues and is associated with HCC metastasis (**A**) miR-199a-5p is significantly decreased in human HCC tissues compared to adjacent-normal hepatic tissues.The expression of miR-199a-5p was normalized to that of U6 small nuclear RNA. (**B, C**) The expression of miR-199a-5p and let-7c was determined by quantitative real-time PCR in HCC cell lines (HepG2, SMMC-7721, MHCC97-H, MHCC97-L, SNU-449 and SNU-398) and the normal human liver cell line L-02. Each sample was analyzed in triplicate and normalized to U6 expression.

In our previous study, we found that let-7c expression was significantly decreased in HCC tissues, and low expression of let-7c is associated with poor differentiation level in HCC. Moreover, in this study, we first examined miR-199a-5p expression in 45 HCC tissues. The results showed that the miR-199a-5p levels were significantly decreased in HCC tissues compared with pair-matched normal hepatic tissues (Figure 1A). We then measured miR-199a-5p and let-7c expression in HCC cell lines. As presented in Figure 1B and 1C, the expression of mature miR-199a-5p and let-7c was lower in cells with high metastatic ability (MHCC-97H, MHCC-97L) than in those with relatively low or no metastatic potential (HepG2, SMMC-7721, SNU-449, SNU-398). To further investigate the correlation between miR-199a-5p expression and the clinicopathological characteristics of the 45 HCC patients. As shown Table 1, low expression of miR-199a-5p was significantly correlated with tumor size (*p* < 0.05), liver envelope invasion (*p* < 0.01). These results suggested that the down-regulation of miR-199a-5p and let-7c might be associated with HCC metastasis.

**Table 1:** Relationship between miR-199a-5p expression and clinicopathological parameters in 45 HCC patients

Parameter	*n* (%)	miR-199a-5p expression		*P* value^a^
		< 0.5-fold	> 0.5-fold	
Age (years)				
≤ 50	20 (44.4)	10	10	
> 50	25 (55.6)	13	12	*P* = 0.894
Gender				
Male	27 (60.0)	15	12	
Female	18 (40.0)	8	10	*P* = 0.465
Hepatitis B virus				
Positive	33 (73.3)	18	15	
Negative	12 (26.7)	5	7	*P* = 0.444
Alpha-fetal protein				
> 400	20 (44.4)	13	7	
≤ 400	25 (55.6)	10	15	*P* = 0.095
Tumour size				
≤ 5 cm	30 (66.7)	12	18	
> 5 cm	15 (33.3)	11	4	***P* = 0.034**
Histological grade				
Moderate	27 (60.0)	12	15	
Poor	18 (40.0)	11	7	*P* = 0.273
Liver envelope invasion				
Positive	19 (42.3)	14	5	
Negative	26 (57.7)	9	17	***P* = 0.009**

^a^Pearson chi-square tests were used. Results were consided statistically significant at *P* < 0.05.

### miR-199a-5p and let-7c cooperatively inhibit HCC cell migration and invasion

**Figure 2:**
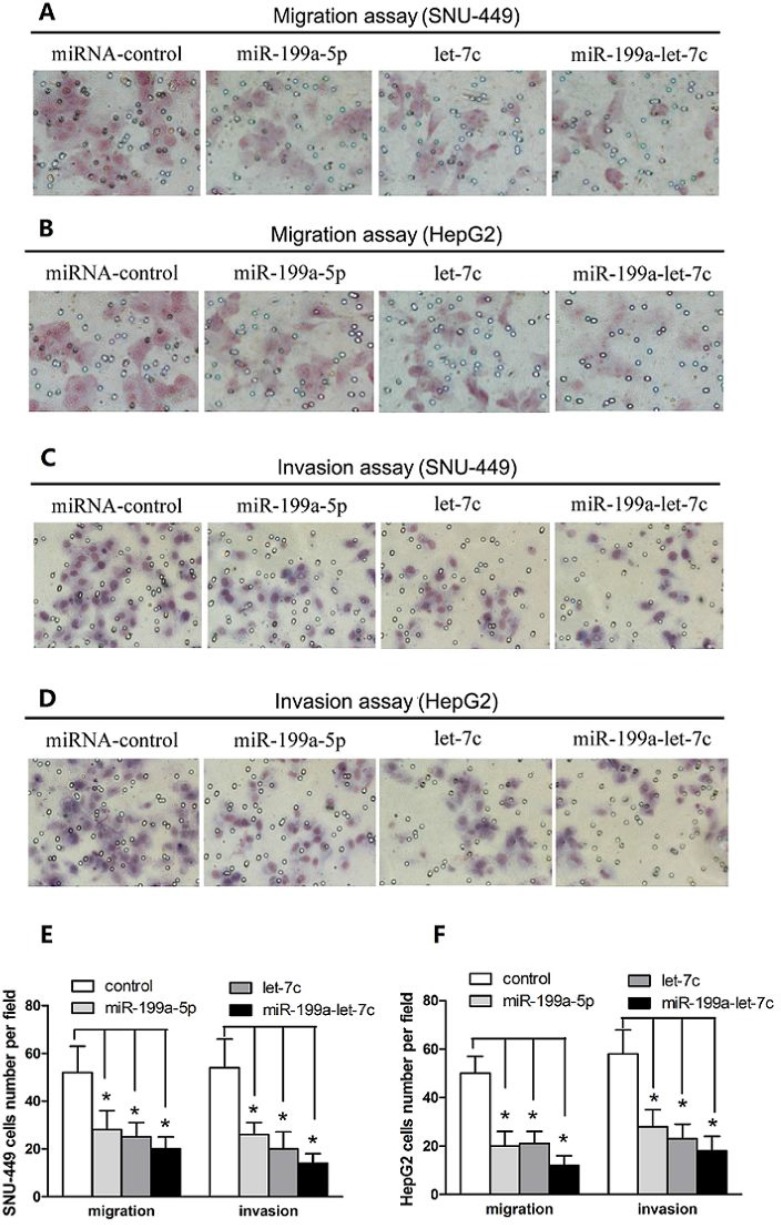
miR-199a-5p and let-7c cooperatively inhibit HCC cell migration and invasion (**A, B**) SNU-449 and HepG2 cells were transfected with miR-199a-5p, let-7c, miRNA negative control, or miR-199a-5p plus let-7c at a final concentration of 50 nM. The ability of SNU-449 and HepG2 cells to migrate was detected by transwell assays. Representative images are presented. (**C, D**) SNU-449 and HepG2 cells were transfected as in (A). The ability of SNU-449 and HepG2 cells to invade was detected by transwell assays. Representative images are presented. (E, F) Transwell migration and invasion assays of SNU-449 and HepG2 cells expressing miR-199a-5p, let-7c, miR-199a-5p and let-7c and negative control. The values are expressed as the mean ± SEM; asterisks indicate significance.

Given that miR-199a-5p and let-7c expression might be associated with the metastatic property of HCC, we assessed whether miR-199a-5p and let-7c play an important role in HCC cell migration and invasion. We first transfected HepG2 and SNU-449 cell lines with miR-199a-5p and let-7c agomirs and confirmed their overexpression through quantitative real-time polymerase chain reaction (PCR) (Supplementary Figure 1). Overexpression of miR-199a-5p and let-7c significantly inhibited the migration of HepG2 and SNU-449 compared with the negative control group (Figure 2A and 2B). In addition, the invasive capacities of HepG2 and SNU-449 transfected with miR-199a-5p and let-7c were substantially less than those of the negative control group (Figure 2C and 2D). Importantly, miR-199a-5p and let-7c exhibited combinatorial effects on the reduction of HCC cell migration and invasion (Figure 2E and 2F). However, the migration and invasion of SMMC-7721 was enhanced when endogenous miR-199a-5p and let-7c were silenced with a miRNA inhibitor (Supplementary Figure 2). Taken together, these results indicated that miR-199a-5p and let-7c cooperatively inhibit HCC cell migration and invasion.

### miR-199a-5p and let-7c significantly suppress luciferase activity cooperatively by directly targeting the 3′-untranslated region (UTR) of MAP4K3

**Figure 3:**
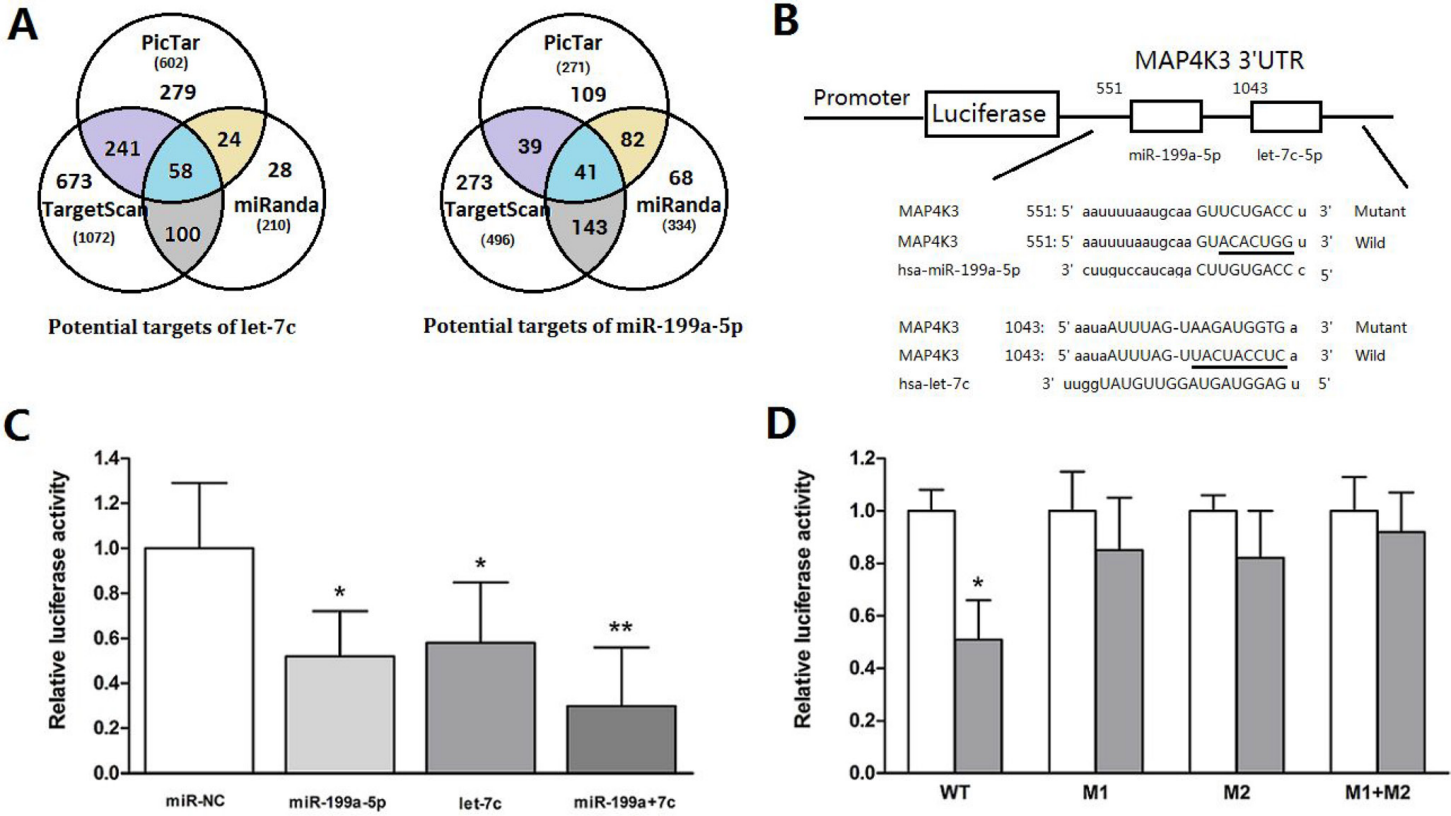
MiR-199a-5p and let-7c significantly suppress luciferase activity cooperatively by directly targeting the 3′-UTR of MAP4K3 (**A**)Schematic representation of the candidate genes predicted by three prediction algorithms. Each labeled circle represents one prediction algorithm with the number of its predicted genes, and the number listed in the overlapping regions of the circles is the number of targets commonly predicted by different algorithms. (**B**) Firefly luciferase reporter vectors containing the MAP4K3 wild-type (pmiR-MAP4K3-3′-UTR-wt) or mutant (pmiR- MAP4K3-3′-UTR–mut) 3′-UTR were generated and co-transfected into HepG2 cells along with miR-199a-5p, let-7c, miR-199a-5p and let-7c or negative control to identify MAP4K3 targets. The 3′UTR of MAP4K3 mRNA contained two complementary sites for the seed region of miR-199a-5p and let-7c. Wild: wild-type; Mut: mutated. The seed sequence is underlined. (**C**) Relative luciferase activity was analyzed after the reporter plasmids or control reporter plasmid was co-transfected with miR-199a-5p or let-7c into HepG2 cells. Representative experiments are presented. Data are shown as the mean ± SEM. Asterisks indicate significance. (**D**) Luciferase reporter vectors, either WT or mutant (M1, M2 and M1+M2), were co-transfected with two miRNAs into HepG2 cells. Luciferase activity was measured 48 h after transfection and normalized to Renilla. Data are shown as the mean ± SEM. Asterisks indicate significance.

To investigate the underlying molecular mechanism by which miR-199a-5p and let-7c inhibit HCC cell migration and invasion, we first used the prediction algorithms TargetScan, PicTar and miRanda to predict potential targets. Fifty-eight candidate genes were predicted to be possible targets of let-7c [19], and forty-one candidate genes were predicted to be possible targets of miR-199a-5p using the three algorithms (Supplementary Table 1). Interestingly, only the MAP4K3 gene was predicted as a possible target of both miR-199a-5p and let-7c (Figure 3A).

To determine whether MAP4K3 is a direct target of miR-199-5p and let-7c, we generated a firefly luciferase reporter vector containing the MAP4K3 3′-UTR (Figure 3B). For luciferase activity assays, HepG2 cells were transfected with the vector along with miRNAs. Relative luciferase activity was mildly reduced by individual miRNAs, whereas transfection with two miRNAs resulted in an approximate 70% decrease, suggesting that these miRNAs act cooperatively (Figure 3C). To further assess whether this reduction was sequence specific, we generated a series of MAP4K3 3′-UTR fragments, including wild-type and mutant fragments that were inserted into the region downstream of the luciferase reporter gene (Figure 3B). The two miRNAs were transfected along with different MAP4K3 vectors into HepG2 cells to test the binding ability of the miRNA combination. The results indicate that the relative luciferase activity of the wild-type was significantly decreased compared with the mutant (Figure 3D), suggesting that miR-199a-5p and let-7c suppress luciferase activity cooperatively by directly targeting the 3′-UTR of MAP4K3.

### MAP4K3 down-regulation requires the cooperation of miR-199a-5p and let-7c, and MAP4K3 promotes HCC cell migration and invasion

**Figure 4:**
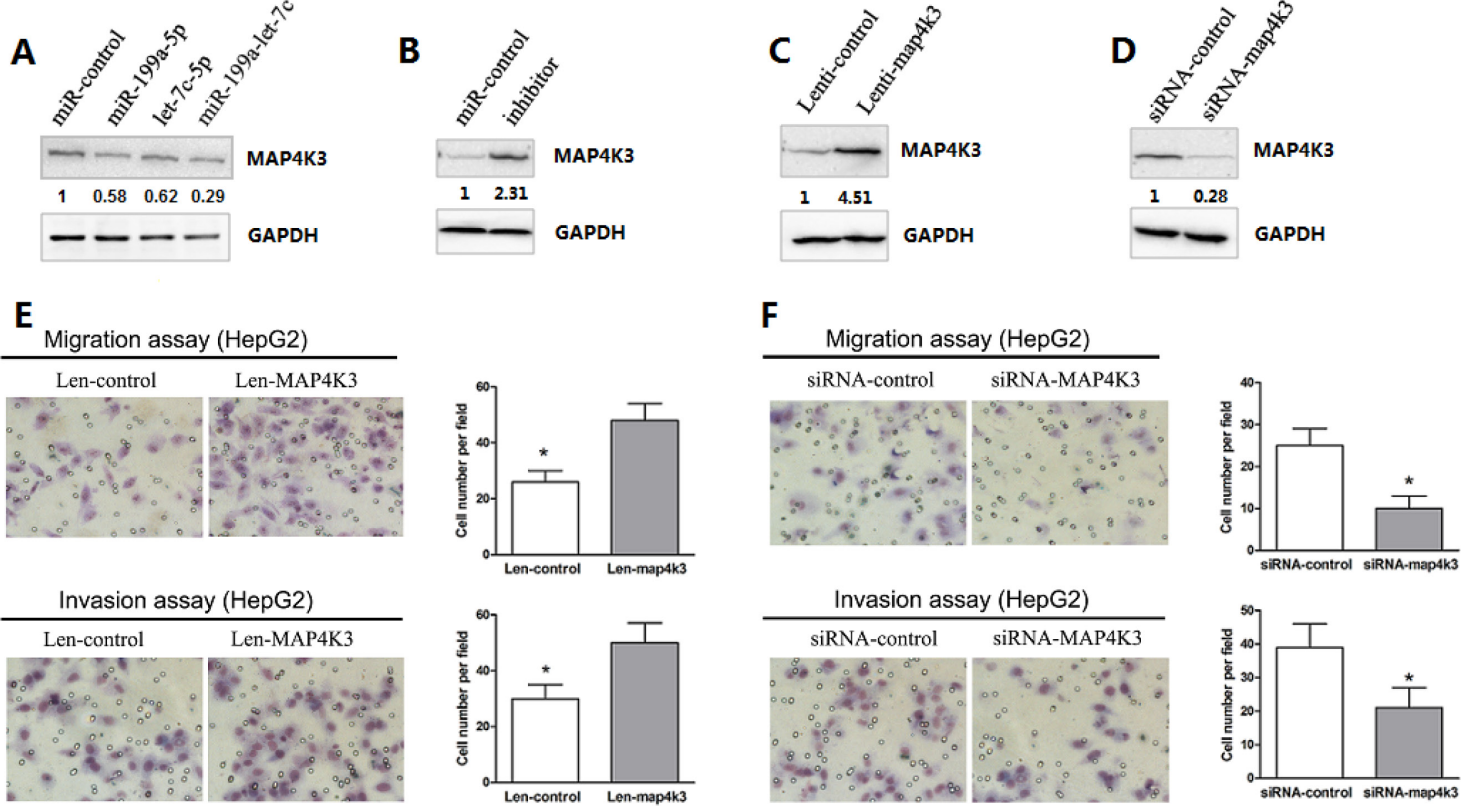
MAP4K3 down-regulation requires the cooperation of miR-199a-5p and let-7c, and MAP4K3 promotes HCC cell migration and invasion (**A, B**) Western blot assays of endogenous MAP4K3 protein levels in HepG2 cells transfected with the miR-199a-5p, let-7c, miR-199a-5p and let-7c agomirs, negative control or miR-199a-5p and let-7c inhibitors. (**C, D**) Western blotting of MAP4K3 protein expression in HepG2 cells infected/transfected with lenti-MAP4K3, lenti-control, MAP4K3-siRNA or negative control-siRNA. (**E, F**) Infection/transfection of HepG2 cells with lenti-MAP4K3, lenti-control, MAP4K3-siRNA or negative control-siRNA was performed to investigate the effects of MAP4K3 on HCC cell migration and invasion. Representative images are presented.

To further verify whether the expression of MAP4K3 is regulated by miR-199a-5p and let-7c, HepG2 cells were transfected with miRNAs, and the expression of each miRNA was confirmed by quantitative PCR analysis 48 h post treatment. As expected, Western blot analysis demonstrated that the endogenous protein level of MAP4K3 was mildly decreased by individual miRNAs. However, when both miRNAs were transfected together, a significant decrease in the MAP4K3 protein level was observed (Figure 4A). This finding was consistent with the luciferase experiments described above. Moreover, the silencing of both let-7c and miR-199a-5p significantly increased the expression of MAP4K3 protein (Figure 4B). However, the mRNA level of MAP4K3 was not significantly influenced by both miRNA agomirs (Supplementary Figure 3), suggesting that MAP4K3 expression was inhibited by miR-199a-5p and let-7c primarily at the post-transcriptional level. Taken together, these results indicated that MAP4K3 down-regulation requires the cooperation of miR-199a-5p and let-7c.

Previous reports have indicated that MAP4K3, a member of the MAP4K family, plays a critical role in migration and invasion [20]. To explore the biological function of MAP4K3 in HCC cells, HepG2 cells were infected with a lentivirus construct containing the MAP4K3 gene or vector alone, and MAP4K3 expression was confirmed by Western blotting (Figure 4C). Remarkably, MAP4K3 overexpression strongly promoted HCC migration and invasion (Figure 4E). In addition, knockdown of endogenous MAP4K3 expression by siRNA in HepG2 cells resulted in a dramatic decrease in HCC cell migration and invasion (Figure 4D and 4F). This phenotype was similar to that induced by the overexpression of miR-199a-5p and let-7c.

### Restoration of MAP4K3 expression promotes miR-199a-5p and let-7c-mediated migration and invasion in HCC cells

**Figure 5:**
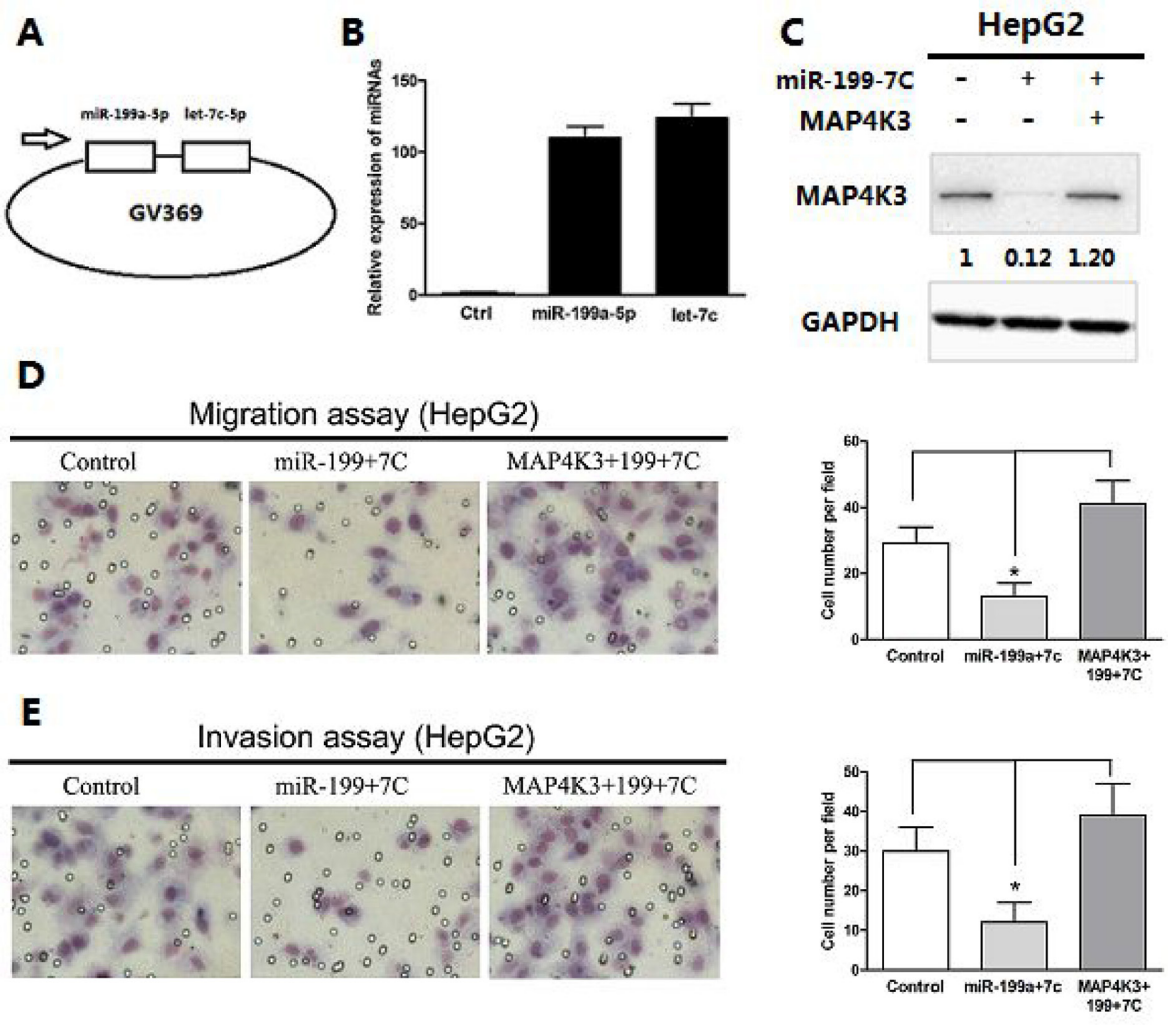
Restoration of MAP4K3 promotes miR-199a-5p- and let-7c-mediated migration and invasion of HCC cells (**A**) Expression vectors were generated by cloning miR-199a-5p and let-7c into a lentiviral vector. (**B**) qRT-PCR assays of mature miR-199a-5p and let-7c expression in HepG2 cells infected with a lentiviral vector expressing miR-199a-5p and let-7c or negative control. (**C**) Western blot analysis of MAP4K3 expression in HepG2 cells or HepG2- miR-199a-5p-let-7c stable cells with or without MAP4K3 reintroduction. (**D**) Transwell migration assays of HepG2 cells or HepG2-miR-199a-5p-let-7c stable cells with or without MAP4K3 reintroduction. Representative images are shown. The values are the mean ± SEM; asterisks indicate significance. (**E**) Transwell invasion assays of HepG2 cells or HepG2-miR-199a-5p-let-7c stable cells with or without MAP4K3 reintroduction.

To improve the delivery efficiency of the two miRNAs and to evaluate the long-term stable expression, we first established a lentiviral expression vector carrying both miR-199a-5p and let-7c (Figure 5A) and confirmed the expression of each miRNA in HepG2 cells (Figure 5B). To further verify that the down-regulation of MAP4K3 is involved in miR-199a-5p- and let-7c-mediated suppression of tumorigenesis, we transfected the coding sequence of MAP4K3 lacking the 3′-UTR into HepG2 stably expressing miR-199a-5p and let-7c, and MAP4K3 expression was confirmed by Western blotting (Figure 5C). As expected, transwell assays demonstrated that the restoration of MAP4K3 expression significantly promoted HCC cell migration and invasiveness initiated by miR-199a-5p and let-7c (Figure 5D and 5E), suggesting that the 3′-UTR region of MAP4K3 is required for the actions of miR-199a-5p and let-7c. This result is consistent with the binding site mutations in the luciferase assay.

### MiR-199a-5p and let-7c increase the sensitivity of HCC cells to sorafenib, which down-regulates MAP4K3 expression

**Figure 6:**
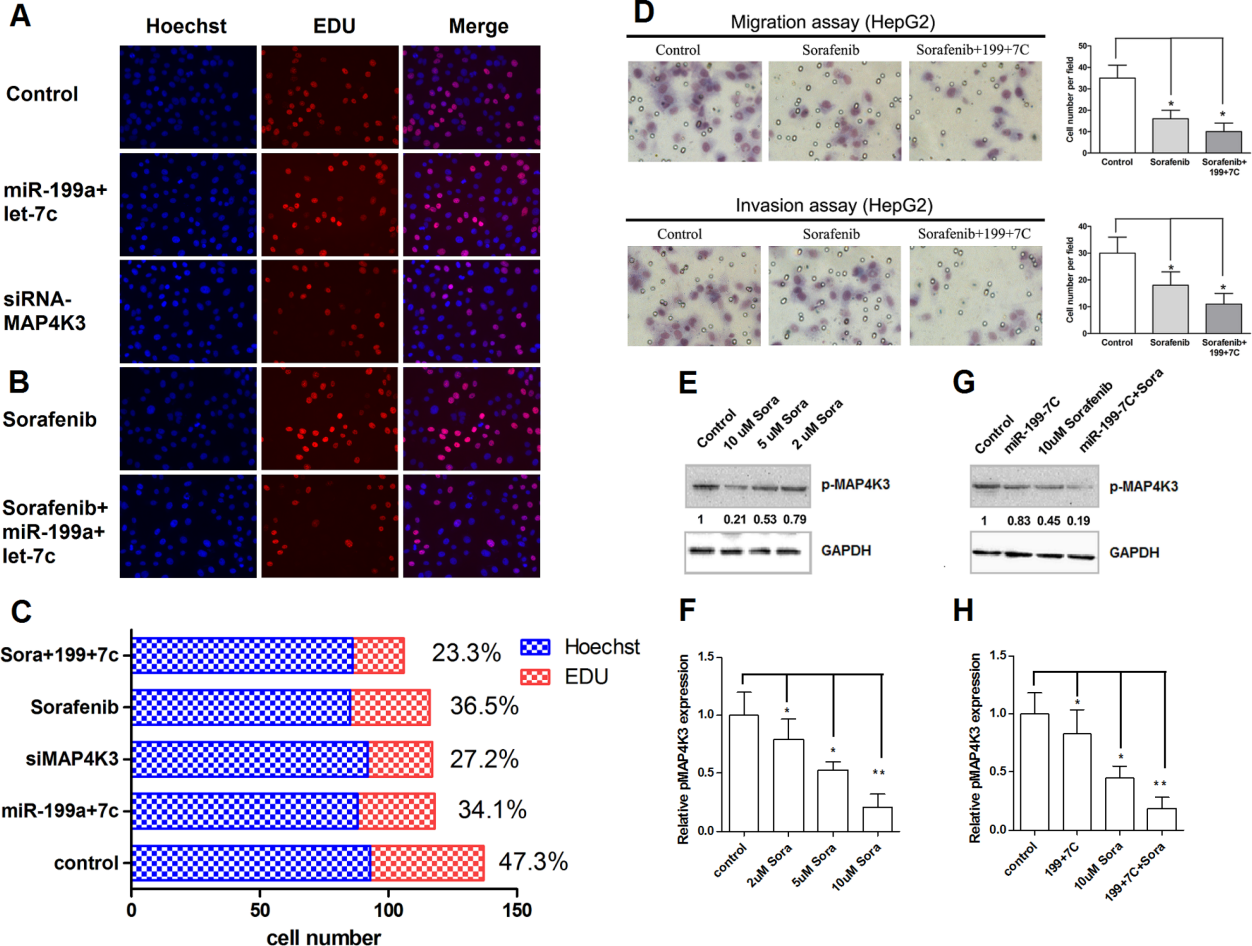
MiR-199a-5p and let-7c increase the sensitivity of HCC cells to sorafenib, which down-regulates MAP4K3 expression (**A**) EDU cell proliferation assays of HepG2 cells transfected miR-199a-5p-let-7c negative control or siRNA-MAP4K3. Representative images are shown. The values are the mean ± SEM; asterisks indicate significance. (**B**) EDU cell proliferation assays of HepG2 cells transfected miR-199a-5p-let-7c or treated with 10 μM sorafenib. Representative images are presented. The values are the mean ± SEM; asterisks indicate significance. (**C**) Cell proliferation Rate of HepG2 cells transfected miR-199a-5p-let-7c, negative control, siRNA-MAP4K3, 10 μM sorafenib or sorafenib plus miR-199a-5p-let-7c. (D) Transwell migration and invasion assays of HepG2 cells transfected with miR-199a-5p-let-7c or treated with 10 μM sorafenib. Representative images are presented. The values are the mean ± SEM; asterisks indicate significance. (**E, F**) HepG2 cells were treated with 2, 5, 10 μM sorafenib or negative control. The levels of p-MAP4K3 protein were detected by Western blotting. The value under each band indicates the relative expression level of p-MAP4K3 compared with GAPDH. (**G, H**) HepG2 cells were treated with the miR-199a-5p and let-7c agomirs, 10 μM sorafenib, sorafenib plus miR-199a-5p and let-7c, or the negative control. The levels of p-MAP4K3 protein were detected by Western blotting. The value under each band indicates the relative expression level of p-MAP4K3 compared with GAPDH.

Previous reports have indicated that microRNAs modulate the sensitivity of cancer cells to chemotherapeutic agents [16, 21]. Therefore, we next tested whether miR-199a-5p and let-7c could sensitize HCC cells to sorafenib. First, we treated HepG2 cells transfected with miR-199a-5p and let-7c with the sorafenib. Cell survival was measured by EDU assays, and cell metastasis was measured by Transwell assays. EDU assays revealed that transfected miR-199a-5p and let-7c caused a significant decrease in cell numbers, a finding that was also observed in cells transfected with siRNA against MAP4K3 (Figure 6A). Sorafenib caused a significant decrease in cell numbers; more importantly, sorafenib caused a further decrease in cell numbers in cells transfected both miR-199a-5p and let-7c (Figure 6B and 6C). In addition, Transwell assays revealed that the transfection of both miR-199a-5p and let-7c in HepG2 cells significantly inhibited cell migration and invasion treatment with sorafenib compared with the controls (Figure 6D).

To understand the underlying mechanism, we measured the level of phospho-MAP4K3 in HepG2 cells treated with different concentrations of sorafenib. The results indicate increased p-MAP4K3 levels in control cells, and these levels were marginally reduced after treatment with 10 μm sorafenib (Figure 6E and 6F). Interestingly, the basal level of p-MAP4K3 was reduced by at least 50% in HepG2 cells treated with 10 μm sorafenib, and these levels were further reduced with increasing concentrations of miR-199a-5p and let-7c (Figure 6G and 6H). Taken together, these results revealed that miR-199a-5p and let-7c expression promotes the growth inhibitory property of sorafenib in HCC cells.

## DISCUSSION

Here, we demonstrate that miR-199a-5p and let-7c are commonly down-regulated in HCC cells and tissues and cooperatively inhibit HCC cell migration and invasion *in vitro*. A direct and functional target of both miR-199a-5p and let-7c was also identified. The target gene, MAP4K3, is frequently overexpressed and promotes metastasis in HCC. Knockdown of endogenous MAP4K3 by siRNA exhibited similar effects to the overexpression of miR-199a-5p and let-7c, whereas overexpression of MAP4K3 abrogated miR-199a-5p- and let-7c-mediated metastasis inhibition. Taken together, these findings indicate that miR-199a-5p and let-7c play fundamental roles in hepatic carcinogenesis, particularly in the process of HCC metastasis.

Metastasis is one of the most important hallmarks of cancer and is the main factor that leads to recurrence and mortality in patients with malignant cancer, particularly with HCC [22]. The long-term survival of HCC patients after curative resection remains low, given the major obstacle of a high recurrence rate, which is mainly due to the spread of intrahepatic metastases. Therefore, the identification of metastatic factors and an understanding of the underlying molecular pathways involved in the progression of metastasis are critical issues.

Recent studies have demonstrated that miRNAs play a fundamental role in the invasion and metastasis of HCC, and their involvement in common cellular pathways make them valuable and comprehensive targets. Yao et al. reported that aberrant expression of miR-30d significantly promotes HCC cell invasion and metastasis through targeting GNAI2 [23]. Tsai et al. suggested that miR-122 affects HCC intrahepatic metastasis partially via the regulation of ADAM17 [24]. Recent findings have demonstrated that miR-199a-5p and let-7c are involved in various biological and pathological processes. For example, Shen et al. reported that the down-regulation of miR-199a-5p not only is highly associated with HCC invasion but also inhibits cell migration and invasion by targeting discoidin domain receptor 1 (DDR1) [17]. The let-7 miRNA family members are widely viewed as tumor suppressors, particularly in lung cancer. Reports have indicated that let-7 inhibits cancer cell proliferation by repressing multiple genes, including RAS, CDK6 and CDC25A [19, 25, 26]. In addition, Zhao et al. reported that let-7c inhibits lung cancer cell migration and invasion by targeting ITGB3 and MAP4K3 [20]. However, the data remain scarce, and we must provide more evidence about the role and underlying molecular mechanism of miR-199a-5p and let-7c in HCC. In our study, we established the role of miR-199a-5p and let-7c in HCC migration and invasion. Moreover, we found that miR-199a-5p and let-7c cooperatively target a single mRNA. This result is consistent with those of previous reports [27, 28]. For example, multiple miRNAs have been recently shown to regulate GRP78 expression in various cancer cells [28]. Importantly, such studies have indicated that miRNA networks tightly control gene expression.

To date, MAPK has been well established as an important mediator of signaling from the cell surface to the nucleus [29]. The MAPK pathway is involved in various cellular functions, including cell proliferation, differentiation and migration [30]. MAP4K3 up-regulation in various tumor types is a major contributor to tumorigenesis, such that Chung-Ping Hsu et al. reported that MAP4K3 overexpression associates with recurrence risk for non-small cell lung cancer [31]. There is date showed that MAP4K3 as a novel apoptosis inducer which modulates cell death via the post-transcriptional regulation of BH3-only proteins in pancreatic cancer [32].We reported that MAP4K3 promotes HCC cell migration and invasion. Previous findings have indicated that miRNA-mediated suppression of protein production potentially occurs at the transcriptional or post-transcriptional level [33, 34]. In this report, we demonstrate that miR-199a-5p and let-7c cooperatively bind to the 3′-UTR of MAP4K3 and dramatically decrease the protein levels of MAP4K3, providing evidence in support of a mechanism for MAP4K3 regulation at the post-transcriptional level. Carlo-Stella demonstrated that the inhibition of the MAPK/ERK pathway using siRNA increases the sensitivity of lymphoma cells to sorafenib [35]. In our study, decreased levels of MAP4K3 achieved using siRNA or transfection of two miRNAs increase the sensitivity of cancer cells to sorafenib, thereby resulting in the inhibition of cell growth, migration and invasion. Therefore, the use of two specific miRNAs combined with sorafenib to target key genes involved in tumorigenesis could provide an exciting avenue for the development of new cancer therapies.

## CONCLUSIONS

This study demonstrates that miR-199a-5p and let-7c cooperatively inhibit HCC cell migration and invasion and that the up-regulation of MAP4K3 subsequently promotes tumorigenesis and therapeutic resistance. Our results suggest that two miRNAs in combination therapy may provide a new and powerful approach for the treatment of metastasis and drug-resistant HCC.

## MATERIALS AND METHODS

### Cell culture and clinical samples

The human HCC cell lines HepG2 (ATCC no. HB-8065), SNU-449 (ATCC no. CRL-2234), and SNU-398 (ATCC no. CRL-2233) were obtained from the American Type Culture Collection (ATCC). SMMC-7721, MHCC97-H, MHCC97-L and the human immortalized liver cell line L-02 were obtained from Shanghai Institutes for Biological Sciences of Chinese Academy of Sciences. HepG2, MHCC-97H and MHCC-97L cells were cultured in Dulbecco's modified Eagle's medium (DMEM). SMMC-7721, SNU-449, SNU-398 and L-02 cell lines were cultured with RPMI 1640. Both media were supplemented with fetal bovine serum to a final concentration of 10% and antibiotics at 37°C with 5% CO2.

Freshly isolated human HCC tissues and paired normal adjacent tissues were obtained from patients undergoing surgery at Zhejiang Provincial People's Hospital during 2008.08-2010.08 with informed consent following the protocols approved by the Ethics Committee of Zhejiang Provincial People's Hospital.

### RNA extraction and quantitative real-time polymerase chain reaction

Total RNA was extracted from HCC cells using TRIzol (Invitrogen, USA) according to the manufacturer's protocol. For miRNA detection, mature miR-199a-5p or let-7c was reverse transcribed with specific RT primers, quantified using a TaqMan probe, and normalized by U6 small nuclear RNA using TaqMan miRNA assays (Assay ID: 000498, Applied Biosystems, CA). MAP4K3 mRNA expression was detected using SYBR Green (Perfect Real Time) (TaKaRa, Otsu, Japan). Glyceraldehyde-3-phosphate dehydrogenase (GAPDH) was used to normalize the MAP4K3 mRNA expression level.The sequences of all primers were listed in Supplementary Table 2. Quantitative real-time PCR was performed using an Applied Biosystems 7500 real-time PCR system (Applied Biosystems). Data analyses were performed using the 2−ΔΔCt method.

### Cell transfection

HepG2, SNU-449, SMMC-7721 or SNU-398 cells were transfected with the miR-199a-5p or let-7c agomir, the negative control or miRNA inhibitor at a final concentration of 50 nM in six-well plates according to the manufacturer's instructions. The miR-199a-5p and let-7c agomirs, miRNA negative control, miRNA inhibitor and negative control were synthesized by Ribobio (Guangzhou, China).

### Lentivirus vector

For the construction of miR-199a-5p and let-7c lentivirus vectors, the pre-miR-199a-5p and pre-let-7c sequences were amplified and cloned into GV369-GFP (System Biosciences, Shanghai, China). The virus particles were harvested 48 h later. GV369-GFP-199a-7c was then co-transfected with the packaging plasmids into HepG2 cells using the Lipofectamine 2000 reagent (Invitrogen).

### EDU assay

Cell proliferation was evaluated using the Cell-Light EdU DNA cell proliferation kit according to the manufacturer's instructions (RiboBio, Guangzhou, China).

### Cell migration and invasion assays

Cellular migration and invasion was assayed using Transwells (Corning Costar Corp). At 48 h post-transfection, 5 × 10^4^ cells were placed on the top chamber of each insert with the noncoated membrane for the migration assay (BD Biosciences, NJ). For the invasion assay, 1 × 10^5^ cells were added to the upper chamber of each insert that was coated with Matrigel (BD Biosciences, NJ).Next, 600 to 800 μl of DMEM or RPMI 1640 containing 10% fetal bovine serum was added into the lower chambers. After 18 h of incubation at 37°C, the cells were fixed with 95% absolute alcohol and stained with 0.1% crystal violet. The cells in the inner chamber were removed, and the cells adhering to the lower membrane were counted and imaged under an inverted microscope (Olympus Corp. Tokyo, Japan) at ×200 magnification.

### miRNA target predictions

Putative miR-199a-5p and let-7c targets were identified using the online predictive algorithms miRanda (http://microrna.sanger.ac.uk), PicTar (http://pictar.mdc-berlin.de/) and TargetScan (http://www.targetscan.org).

### Luciferase reporter assays

MAP4K3 was selected as a common potential target of miR-199a-5p and let-7c. Both the miR-199a-5p and let-7c binding sites at the MAP4K3 3′-UTR were amplified by PCR from HEK-293 cell genomic DNA and cloned into the XhoI and NotI sites downstream of the luciferase reporter gene in the pmiR-Check-REPORT vector. Three mutant constructs were generated using the KOD-Plus-mutagenesis kit (TOYOBO, Osaka, Japan). All constructs were verified by DNA sequencing. HepG2 cells were co-transfected with pmiR MAP4K3-3′-UTR-wt or pmiR- MAP4K3-3′-UTR-mut reporters along with either the miR-199a-5p and let-7c agomirs or negative control in 96-well plates using Lipofectamine 2000. Luciferase activity was measured using the dual luciferase assay system (Promega, Heidelberg, Germany) 48 h post transfection. Renilla luciferase was used as an internal control; the firefly luciferase activity of each sample was normalized to the activity of Renilla luciferase. The experiment was performed independently in triplicate.

### Western blotting

Total protein from the transfected or infected cells was extracted using standard protocols. The target protein levels were detected using primary antibodies against MAP4K3, p-MAP4K3, and GAPDH (Abcam, Cambridge, UK). The values of these proteins were normalized for the corresponding values of GAPDH. Band signals were acquired in the linear range of the scanner and analyzed using QUANTITY ONE software (Bio-Rad, Hercules, CA, USA).

### Statistical analysis

Statistical analyses were performed using SPSS17.0 software. The results are presented as the mean ± standard error of at least three independent experiments. *P* < 0.05 was regarded as statistically significant and is represented as *.

## SUPPLEMENTARY MATERIALS FIGURES AND TABLES



## References

[1] El-Serag HB (2012). Epidemiology of viral hepatitis and hepatocellular carcinoma. Gastroenterology.

[2] Forner A, Llovet JM, Bruix J (2012). Hepatocellular carcinoma. Lancet.

[3] Bruix J, Sherman M (2011). Management of hepatocellular carcinoma: an update. Hepatology.

[4] Yang HI, Yeh SH, Chen PJ, Iloeje UH, Jen CL, Su J, Wang LY, Lu SN, You SL, Chen DS, Liaw YF, Chen CJ (2008). Associations between hepatitis B virus genotype and mutants and the risk of hepatocellular carcinoma. J Natl Cancer Inst.

[5] Bruno S, Crosignani A, Maisonneuve P, Rossi S, Silini E, Mondelli MU (2007). Hepatitis C virus genotype 1b as a major risk factor associated with hepatocellular carcinoma in patients with cirrhosis: a seventeen-year prospective cohort study. Hepatology.

[6] He L, Hannon GJ (2004). MicroRNAs: small RNAs with a big role in gene regulation. Nat Rev Genet.

[7] Budhu A, Jia HL, Forgues M, Liu CG, Goldstein D, Lam A, Zanetti KA, Ye QH, Qin LX, Croce CM, Tang ZY, Wang XW (2008). Identification of metastasis-related microRNAs in hepatocellular carcinoma. Hepatology.

[8] Han ZB, Zhong L, Teng MJ, Fan JW, Tang HM, Wu JY, Chen HY, Wang ZW, Qiu GQ, Peng ZH (2012). Identification of recurrence-related microRNAs in hepatocellular carcinoma following liver transplantation. Mol Oncol.

[9] Gu H, Guo X, Zou L, Zhu H, Zhang J (2013). Upregulation of microRNA-372 associates with tumor progression and prognosis in hepatocellular carcinoma. Mol Cell Biochem.

[10] Xu J, Zhu X, Wu L, Yang R, Yang Z, Wang Q, Wu F (2012). MicroRNA-122 suppresses cell proliferation and induces cell apoptosis in hepatocellular carcinoma by directly targeting Wnt/β-catenin pathway. Liver Int.

[11] Najafi Z, Sharifi M, Javadi G (2015). Degradation of miR-21 induces apoptosis and inhibits cell proliferation in human hepatocellular carcinoma. Cancer Gene Ther.

[12] Lian J, Jing Y, Dong Q, Huan L, Chen D, Bao C, Wang Q, Zhao F, Li J, Yao M, Qin L, Liang L, He X (2016). miR-192, a prognostic indicator, targets the SLC39A6/SNAIL pathway to reduce tumor metastasis in human hepatocellular carcinoma. Oncotarget.

[13] Johnson SM, Grosshans H, Shingara J, Byrom M, Jarvis R, Cheng A, Labourier E, Reinert KL, Brown D, Slack FJ (2005). RAS is regulated by the let-7 microRNA family. Cell.

[14] Takamizawa J, Konishi H, Yanagisawa K, Tomida S, Osada H, Endoh H, Harano T, Yatabe Y, Nagino M, Nimura Y, Mitsudomi T, Takahashi T (2004). Reduced expression of the let-7 microRNAs in human lung cancers in association with shortened postoperative survival. Cancer Res.

[15] Han HB, Gu J, Zuo HJ, Chen ZG, Zhao W, Li M, Ji DB, Lu YY, Zhang ZQ (2012). Let-7c functions as a metastasis suppressor by targeting MMP11 and PBX3 in colorectal cancer. J Pathol.

[16] Shimizu S, Takehara T, Hikita H, Kodama T, Miyagi T, Hosui A, Tatsumi T, Ishida H, Noda T, Nagano H, Doki Y, Mori M, Hayashi N (2010). The let-7 family of microRNAs inhibits Bcl-xL expression and potentiates sorafenib-induced apoptosis in human hepatocellular carcinoma. J Hepatol.

[17] Shen Q, Cicinnati VR, Zhang X, Iacob S, Weber F, Sotiropoulos GC, Radtke A, Lu M, Paul A, Gerken G, Beckebaum S (2010). Role of microRNA-199a-5p and discoidin domain receptor 1 in human hepatocellular carcinoma invasion. Mol Cancer.

[18] Zhu XM, Wu LJ, Xu J, Yang R, Wu FS (2011). Let-7c microRNA expression and clinical significance in hepatocellular carcinoma. J Int Med Res.

[19] Zhu X, Wu L, Yao J, Jiang H, Wang Q, Yang Z, Wu F (2015). MicroRNA let-7c Inhibits Cell Proliferation and Induces Cell Cycle Arrest by Targeting CDC25A in Human Hepatocellular Carcinoma. PLoS One.

[20] Zhao B, Han H, Chen J, Zhang Z, Li S, Fang F, Zheng Q, Ma Y, Zhang J, Wu N, Yang Y (2014). MicroRNA let-7c inhibits migration and invasion of human non-small cell lung cancer by targeting ITGB3 and MAP4K3. Cancer Lett.

[21] Sun C, Li N, Yang Z, Zhou B, He Y, Weng D, Fang Y, Wu P, Chen P, Yang X, Ma D, Zhou J, Chen G (2013). MiR-9 regulation of BRCA1 and ovarian cancer sensitivity to cisplatin and PARP inhibition. J Natl Cancer Inst.

[22] Hanahan D, Weinberg RA (2011). Hallmarks of cancer: the next generation. Cell.

[23] Yao J, Liang L, Huang S, Ding J, Tan N, Zhao Y, Yan M, Ge C, Zhang Z, Chen T, Wan D, Yao M, Li J (2010). MicroRNA-30d promotes tumor invasion and metastasis by targeting Galphai2 in hepatocellular carcinoma. Hepatology.

[24] Tsai WC, Hsu PW, Lai TC, Chau GY, Lin CW, Chen CM, Lin CD, Liao YL, Wang JL, Chau YP, Hsu MT, Hsiao M, Huang HD (2009). MicroRNA-122, a tumor suppressor microRNA that regulates intrahepatic metastasis of hepatocellular carcinoma. Hepatology.

[25] Johnson SM, Grosshans H, Shingara J, Byrom M, Jarvis R, Cheng A, Labourier E, Reinert KL, Brown D, Slack FJ (2005). RAS is regulated by the let-7 microRNA family. Cell.

[26] Johnson CD, Esquela-Kerscher A, Stefani G, Byrom M, Kelnar K, Ovcharenko D, Wilson M, Wang X, Shelton J, Shingara J, Chin L, Brown D, Slack FJ (2007). The let-7 microRNA represses cell proliferation pathways in human cells. Cancer Res.

[27] Mavrakis KJ, Leslie CS, Wendel HG (2011). Cooperative control of tumor suppressor genes by a network of oncogenic microRNAs. Cell Cycle.

[28] Su SF, Chang YW, Andreu-Vieyra C, Fang JY, Yang Z, Han B, Lee AS, Liang G (2013). miR-30d, miR-181a and miR-199a-5p cooperatively suppress the endoplasmic reticulum chaperone and signaling regulator GRP78 in cancer. Oncogene.

[29] Beeram M, Patnaik A, Rowinsky EK (2005). Raf: a strategic target for therapeutic development against cancer. J Clin Oncol.

[30] Cargnello M, Roux PP (2011). Activation and function of the MAPKs and their substrates, the MAPK-activated protein kinases. Microbiology and Molecular Biology.

[31] Hsu CP, Chuang HC, Lee MC, Tsou HH, Lee LW, Li JP, Tan TH (2016). GLK/MAP4K3 overexpression associates with recurrence risk for non-small cell lung cancer. Oncotarget.

[32] Lam D, Dickens D, Reid EB, Loh SH, Moisoi N, Martins LM (2009). MAP4K3 modulates cell death via the post-transcriptional regulation of BH3-only proteins. Proc Natl Acad Sci USA.

[33] Carvalheira G, Nozima BH, Cerutti JM (2015). microRNA-106b-mediated down-regulation of C1orf24 expression induces apoptosis and suppresses invasion of thyroid cancer. Oncotarget.

[34] Song R, Walentek P, Sponer N, Klimke A, Lee JS, Dixon G, Harland R, Wan Y, Lishko P, Lize M, Kessel M, He L (2014). miR-34/449 miRNAs are required for motile ciliogenesis by repressing cp110. Nature.

[35] Carlo-Stella C, Locatelli SL, Giacomini A, Cleris L, Saba E, Righi M, Guidetti A, Gianni AM (2013). Sorafenib inhibits lymphoma xenografts by targeting MAPK/ERK and AKT pathways in tumor and vascular cells. PLoS One.

