# Supplementary material for: MiR-199a-5p and let-7c cooperatively inhibit migration and invasion by targeting MAP4K3 in hepatocellular carcinoma
Source: Oncotarget. 2017 Jan 13;8(8):13666–77. doi: 10.18632/oncotarget.14623 (PMC5355128; doi:10.18632/oncotarget.14623)
Supplement: Supplementary file 1 [file oncotarget-08-13666-s001.pdf]

MiR-199a-5p and let-7c cooperatively inhibit migration and invasion by targeting MAP4K3 in hepatocellular carcinoma

Supplementary Materials

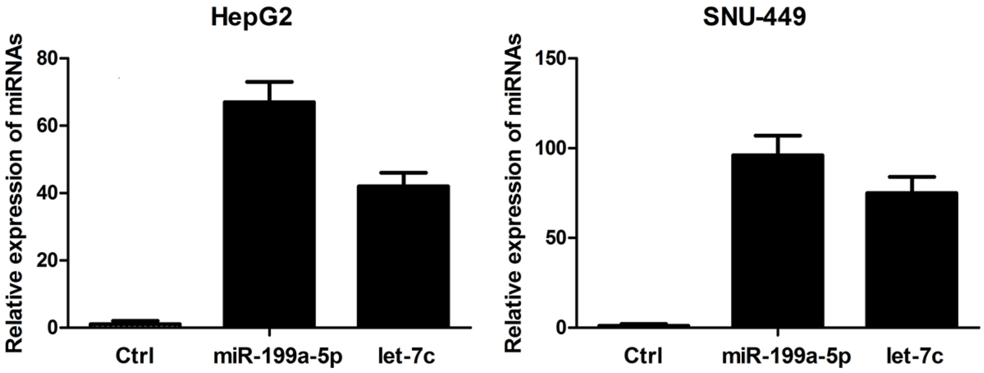

Supplementary Figure 1: Expression of miR-199a-5p and let-7c in HCC cells after transfection. HepG2 and SNU-449 HCC cells were transfected with the miR-199a-5p or let-7c agomir at a final concentration of 50 nM. Expression of miR-199a-5p and let-7c was determined using quantitative real-time PCR 48 h post-transfection.

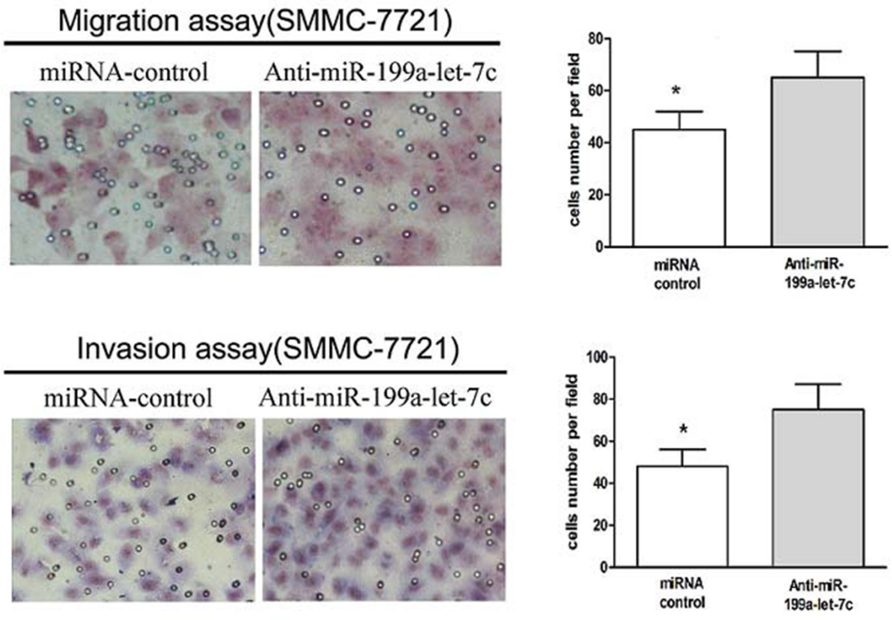

Supplementary Figure 2: MiR-199a-5p and let-7c cooperatively inhibit HCC cell migration and invasion. SMMC-7721 cells were transfected with miR-199a-5p and let-7c inhibitors or negative control at a final concentration of 50 nM. The migration and invasion of SMMC-7721 were detected by transwell assays. Representative images are shown on the left. The values shown are expressed as the mean ± SEM; asterisks indicate significance.

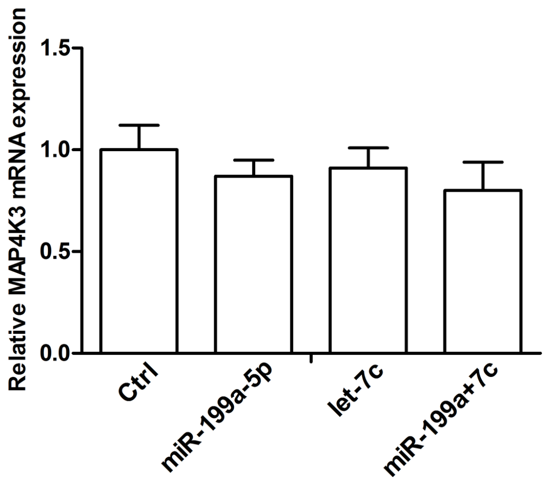

Supplementary Figure 3: Real-time PCR assay of MAP4K3 mRNA expression in HepG2 cells transfected with the miR-199a-5p, let-7c, miR-199a-5p or let-7c agomirs or negative control.

Supplementary Table 1: Forty-one candidate genes were predicted to be the possible targets of miR-199a-5p by the three algorithms

Target gene	Gene description
APPBP2	amyloid beta precursor protein binding protein 2
ARHGAP12	Rho GTPase activating protein 12
ARHGAP21	Rho GTPase activating protein 21
ATXN7	ataxin 7
CCNL1	cyclin L1
CELSR1	cadherin, EGF LAG seven-pass G-type receptor 1
EPB41L1	erythrocyte membrane protein band 4.1-like 1
EPHA7	EPH receptor A7
FLRT3	fibronectin leucine rich transmembrane protein 3
FZD4	frizzled class receptor 4
GRB10	growth factor receptor-bound protein 10
HIF1A	hypoxia inducible factor 1, alpha subunit
HLF	hepatic leukemia factor
HSPA5	heat shock 70kDa protein 5 (glucose-regulated protein)
JUNB	jun B proto-oncogene
KIAA0355	KIAA0355
KL	klotho
KPNA4	karyopherin alpha 4 (importin alpha 3)
LAMC1	laminin, gamma 1 (formerly LAMB2)
LIN7C	lin-7 homolog C (C. elegans)
MAB21L1	mab-21-like 1 (C. elegans)
MAP3K11	mitogen-activated protein kinase kinase kinase 11
**MAP4K3**	mitogen-activated protein kinase kinase kinase kinase 3
MPP5	membrane protein, palmitoylated 5
MYH9	myosin, heavy chain 9, non-muscle
NLK	nemo-like kinase
NPAS2	neuronal PAS domain protein 2
PAN3	PAN3 poly(A) specific ribonuclease subunit
PLXND1	plexin D1
PPARGC1A	peroxisome proliferator-activated receptor gamma, coactivator 1 alpha
PPP1R2	protein phosphatase 1, regulatory (inhibitor) subunit 2
RAD23B	RAD23 homolog B (S. cerevisiae)
RANBP2	RAN binding protein 2
RNF38	ring finger protein 38
RORB	RAR-related orphan receptor B
SACS	sacsin molecular chaperone
SLC24A3	solute carrier family 24
SMARCD1	SWI/SNF related, matrix associated, actin dependent regulator of chromatin, subfamily d, member 1
STK4	serine/threonine kinase 4
TAB3	TGF-beta activated kinase 1/MAP3K7 binding protein 3
WDTC1	WD and tetratricopeptide repeats 1

Supplementary Table 2: Primer sequences for qPCR and miRNA agomir sequences

No.	Name		Sequence
1	MAP4K3 (qPCR)	Forward	5′- GCAAAGCCATCCCAAGTT -3′
		Reverse	5′- GTGCCTCTATGTTCATTCTGTT-3′
2	GAPDH (qPCR)	Forward	5′- GCTGAGAACGGGAAGCTTGT -3′
		Reverse	5′- GCCAGGGGTGCTAAGCAG -3′
3	mir-199a-let-7c	Forward	5′-GAGGATCCCCGGGTACCGGTTGGTGGTTTCCTTGGCTG-3′
		Reverse	5′-CACACATTCCACAGGCTAGGATACTCATGACACATTAC-3′
4	miR-199a-5p agomir		5′-CCCAGUGUUCAGACUACCUGUUC-3′
5	miR-199a-5p inhibitor		5′-CACCGUUUGUAGCAACUUGUGG-3′
6	let-7c agomir		5′-UGAGGUAGUAGGGUUGUAUGGUU-3′
7	let-7c inhibitor		5′-AACCAUACAACCUACUACCUCA-3′
8	negative control		5′-CAGUACUUUUGUGUAGUACAA-3′

mir-199a-let-7c: miR-199a-5p and let-7c recombinant plasmid construct.
